# Inferior Vena Cava Filters: An Overview

**DOI:** 10.14797/mdcvj.1346

**Published:** 2024-05-16

**Authors:** Paul Haddad, Jasmine Peng, Madeline Drake, Maham Rahimi

**Affiliations:** 1 Houston Methodist Hospital, Houston, Texas, US; 2 Texas A&M College of Medicine, Bryan, Texas, US; 3 Methodist DeBakey Heart & Vascular Center, Houston, Texas, US

**Keywords:** vascular surgery, inferior vena cava, inferior vena cava filter, deep vein thrombosis

## Abstract

For patients with existing venous thromboembolisms (VTEs), anticoagulation remains the standard of care recommended across multiple professional organizations. However, for patients who developed a deep venous thrombosis (DVT) and/or a pulmonary embolism and cannot tolerate anticoagulation, inferior vena cava (IVC) filters must be considered among other alternative treatments. Although placement of a filter is considered a low-risk intervention, there are important factors and techniques that surgeons and interventionalists should be aware of and prepared to discuss. This overview covers the basics regarding the history of filters, indications for placement, associated risks, and techniques for difficult removal.

## History and Mechanism

Pulmonary embolism (PE), a feared and fatal complication of surgery, is the third most common cause of cardiovascular death worldwide.^1,2,3^ Most pulmonary emboli is known to originate from lower extremity deep vein thromboses (DVT),^1,2,4^ and patients with initial PE have higher rates of recurrence.^2^ Presently, the mainstay of treatment and prevention is anticoagulation.^4,5^ However, for patients with contraindications to anticoagulation or in those who experience repeat PE despite adequate anticoagulation, further intervention is necessary.^4,5^ For these patients, placement of an IVC filter is a significant therapeutic option that has been recommended across multiple professional organizations.^6,7,8^

The incidence of leg DVT has been reported to range from 45 to 117 per 100,000 people per year, and the reported incidence of PE with existing DVT has ranged from 29 to 78 per 100,000 people per year.^9^ Since venous thromboembolism (VTE), which encompasses both DVT and PE, is a disease of old age, the incidence of DVT and PE increases with age for men and women alike.^9^ According to one comprehensive review, the risk of death is 18-times greater for patients with PE than for those with DVT alone, and PE is considered an independent predictor of mortality for up to 3 months after discovery.^9^ It is estimated that up to 30% of patients will experience VTE recurrence within 10 years.^9^ Of patients with preexisting DVTs, those with lower extremity DVT (LEDVT) are more at risk for subsequent PE development than those with upper extremity DVT (UEDVT).^10^

The idea of interrupting lower extremity venous return as a means of preventing PE began in the 1930s and 1940s with femoral vein ligation. This technique was minimally effective with significant associated morbidity, prompting the transition to partial vena cava occlusion devices by the 1960s.^3,5^ The most notable of these devices was the Adams-Deweese clip, which was placed via laparotomy external to the IVC to restrict cross-sectional area while maintaining adequate blood flow.^3,4,5^ Two of the first permanent devices, the Greenfield IVC filter and the Mobin-Uddin umbrella filter, were both introduced for general use in 1973 and inserted through venotomy.^5^ Although the Mobin-Uddin is no longer available, the Greenfield stainless steel filter remains on the market. It has become the longest used and most extensively evaluated filter, introduced as a percutaneous placement technique by 1984.^5^ Transvenous devices are more commonly used today due to their minimally invasive approach.^5^ The common femoral vein or jugular vein are most often accessed percutaneously under ultrasound guidance and the filter is placed in the infrarenal IVC.

By 2004, the United States (US) Food and Drug Administration (FDA) had approved a retrievable filter for use.^11^ This resulted in an initial uptick in filter placement as indications for use expanded.^6,11^ The reasoning behind this expansion was founded on the idea that retrievable filters would decrease the risk of thrombotic complications that came with extended use.^12^ However, some recent studies comparing patient outcomes in permanent versus retrievable filter use have shown no difference in performance or complication rates between the two, perhaps due to low retrieval rates (mean = 36%).^12^

## Indications

Indications for IVC filter placement can be split into three main categories: classic, expanded, and prophylactic.^6,13^ The classic indication for placement is the presence of a VTE with an absolute or relative contraindication to anticoagulation.^6,13^ Absolute contraindications include active bleeding, recent intracranial hemorrhage, platelet count < 50,000/uL, and planned procedure with high bleeding risk.^5,7^ Relative contraindications include recurrent gastrointestinal bleeds, presence of intracranial or spinal tumors, and platelet count < 150,000/uL.^7^ Professional societies such as the American Heart Association, the American College of Radiology, and the Society of Interventional Radiology agree on these indications (Table 1).^6,7,13^

**Table 1:** Indications for vena cava filter use from professional societies.^6,8^ DVT/PE: deep vein thrombosis/pulmonary embolism; VTE: venousthromboembolism; IVC: inferior vena cava


INDICATIONS		AMERICAN HEART ASSOCIATION (2011)	AMERICAN COLLEGE OF CHEST PHYSICIANS (2016)	AMERICAN COLLEGE OF RADIOLOGY/SOCIETY OF INTERVENTIONAL RADIOLOGY (2023)

Classic	Acute proximal DVT/PE with contraindication to anticoagulation	Class I, level C	Grade 1B	Usually appropriate

	VTE recurrence despite anticoagulation	Class IIa, level C		Usually appropriate

	Propagation/progression of VTE despite anticoagulation or inability to maintain anticoagulation			Usually appropriate

Expanded	Massive PE with residual DVT in high-risk patients	Class IIb, level C		May be appropriate

	Free-floating iliofemoral or IVC thrombus			May be appropriate

	Severe cardiopulmonary disease/poor reserve and DVT or PE	Class IIb, level C		May be appropriate

Prophylactic	Prophylactic placement in high-risk trauma patients who cannot be anticoagulated			May be appropriate


Though a true prospective randomized controlled trial testing the efficacy of IVC filters is unlikely to occur (since it would be unethical to require a control group not to receive intervention in the setting of VTE), a few retrospective and observational studies show a reduction in fatal PEs with filter placement.^6,8,13^ Also, general consensus is that patients who have failed or experienced a major complication to anticoagulation also fall under the classic indication for IVC filter placement.^7,14^ Examples include recurrent VTE, progression of a DVT despite adequate anticoagulation therapy, and patients who experience major bleeding while on anticoagulation.^7^

Expanded indications encompass patients with VTE who also are receiving anticoagulation therapy and remain high risk.^6,13^ These indications largely emerged following the development of retrievable filters in the late 1990s and resulted in an initial increase in placement.^6^ Expanded indications include use as an adjunct in patients with massive or high-risk PE with concurrent DVT, iliocaval or large free-floating proximal DVT, difficulty maintaining therapeutic anticoagulation, poor compliance with anticoagulation, and high risk for complications associated with anticoagulation, such as frequent falls, presence of cancer, alcohol abuse, renal or liver failure, and diabetes, among others.^6,13,15,16^

Two randomized controlled trials, Prevention du Risque d’Embolie Pulmonaire par Interruption Cave (PREPIC1 and PREPIC2) published in 1998 and 2005 respectively, evaluated the efficacy of using filters on top of adequate anticoagulation.^14,17^ In PREPIC1, 400 patients with proximal DVTs and at high risk for PE were split into two treatment groups: anticoagulation alone or anticoagulation with permanent filter placement. The study found that patients with concurrent filter placement plus anticoagulation had a significantly lower risk of PE after 12 days, although no significant difference was seen at 2 years.^14^ PREPIC2 assessed the efficacy of retrievable IVC filters specifically.^17^ All 399 patients were anticoagulated for 6 months, then the treatment group received a removable IVC filter that was then removed after 3 months.^17^ At 3 and 6 months after removal, the two groups had no significant differences in recurrent PE and mortality.^17^ Because of the limited evidence to support expanded indications for IVC filters, professional societies differ in their recommendations.^6,7,13^ However, permanent IVC filters are generally no longer recommended due to the PREPIC1 study finding increased rates of DVT and no improvement in mortality despite decreased rates of PE.^8,14^

Lastly, the prophylactic indication describes patients who do not have VTE but are at high risk for developing one.^6,7,13^ These include patients who have experienced major trauma, who are scheduled to undergo major orthopedic surgery, and who have underlying medical conditions that predispose to VTE.^6,8^ The use of IVC filters for prophylaxis has increased since the incorporation of retrievable filters in practice.^8^ Since trauma patients are typically immobile, hypercoagulable, and have endothelial injury, they are at particularly high risk for VTE.^6^ However, data is conflicting on whether prophylactic filters reduce symptomatic and fatal PE in trauma patients who cannot receive anticoagulation. Practice varies widely between institutions, and professional society recommendations also vary.

Suprarenal placement of IVC filters is reserved for indications such as extensive infrarenal IVC thrombosis, renal or gonadal vein thrombosis, pregnant women with DVT, an existing infrarenal IVC filter that has thrombosed with the thrombus extending superiorly, and congenital IVC anomalies such as duplicated IVC.^18,19^ Several retrospective and comparative studies have shown that suprarenal placement of IVC filters does not carry an additional risk of complications during use or retrieval.^18^

### Superior Vena Cava Placement

Despite increasing incidence of UEDVT with the development and use of peripherally inserted central lines, the overall incidence of UEDVT remains low, accounting for only 4% to 6% of all DVTs.^20^ Placing filters in the superior vena cava (SVC) to prevent PE in patients with known UEDVT remains controversial and is considered an off-label use, as currently no FDA-approved SVC filter exists.^10,20^ One comprehensive literature review of 21 publications describing SVC filter use found only a 5.6% incidence of PE in patients with UEDVT and a mortality rate from PE of 0.7%, which they concluded was not enough evidence to demonstrate significant risk of UEDVTs.^10^ Another single-center 10-year retrospective review came to a similar conclusion and further identified a significant rate of complications, such as filter misplacement, SVC perforation and/or thrombosis, recurrent pneumothorax, and filter tilting.^20^ Leg perforation, when a strut or leg of the filter punctures through the venous wall, is a known complication of IVC filters that is generally asymptomatic.^20^ However, this complication becomes much more problematic with SVC placement due to the close proximity of the aorta, duodenum, kidneys, and vertebral bodies.^20^ In this study, the leg perforation was found in 10 of 24 patients with available post-filter imaging, although the authors admit that a high lost-to-follow-up rate made it difficult to draw conclusions about the significance of this complication.^20^ Because of insufficient evidence to suggest that the benefits of placing an SVC filter outweigh the risks, most studies do not advocate for their use.^10,20^

### Specific Subpopulations

#### Bariatric Patients

Following sepsis and anastomotic leak, VTE is the next leading cause of death postoperatively in bariatric surgery patients.^21^ This is because morbid obesity is considered a separate risk factor for VTE.^15,21^ Currently, anticoagulation is still the primary method used for prophylaxis, although this setting creates unique dosing challenges since weight-based calculations are not always accurate in patients with extremely high body mass indexes.^15^ Currently, prophylactic IVC filter placement is not recommended to prevent VTE or PE since placement has not been shown to significantly reduce rates of either thrombotic complication.^6,15,21,22^ Some studies have shown increased rates of DVT and morbidity with prophylactic filter placement.^6,8,15,21^

#### Pediatric Patients

Comprehensive data on IVC filter use, outcomes, and guidelines in the pediatric population is sparse. One single-center study following 59 patients over 10 years showed that most pediatric filter placement was for prophylaxis in the trauma setting.^23^ Their findings of a high filter complication rate (16.9%) and low filter retrieval rate (20.3%) led to the conclusion that the evidence to support prophylactic IVC filter placement in pediatrics is weak. A second retrospective multicenter cohort study found that the rate of pediatric IVC filter placement did not increase over their 8-year study period, unlike the rate in adult populations, and that filter placement remained a rather rare event, with a mean incidence of 6 per 100,000 admissions.^24^ Contrasting the results of the single-center study, this study demonstrated that prophylactic placement was an uncommon occurrence, as 76% of patients receiving filters had a preexisting VTE.^24^

#### Cancer Patients

The fact that cancer is a prothrombotic state that increases risk for VTE is well established, and the mechanism is hypothesized to include a combination of factors such as procoagulant and inflammatory cytokine production by the tumor, increased expression of intrinsic factor, immobilization, and endothelial damage/vessel wall changes.^8,20^ One group found that although IVC filters are commonly used for PE management in this population, the efficacy of this practice is uncertain since VTE recurrence rates remain high. The most common indication for placing a temporary filter is active bleeding.^20^

## Risks

Obtaining consent for IVC filter placement can often be a prolonged discussion regarding preoperative, intraoperative, and postoperative risks. During the preoperative discussion, it is important to review the benefits of timely progression to the operating room or interventional suite as the patient is not anticoagulated and therefore is at elevated risk of thromboembolism. Delay in care should be avoided and IVC filter placement should be done as soon as possible. Intraoperative risks include standard surgical risks involved with percutaneous access such as bleeding, infection, pain, and injury to nearby structures. Regarding filter placement, there is a risk of misdeployment that may require removal and replacement. Additionally, the filter may embolize or migrate, which requires further procedures to retrieve it and, in rare instances, an open surgical intervention. Finally, and most importantly, are the rare postoperative risks including filter fracture, embolization, vessel penetration, and IVC thrombosis.

Embolization most often occurs during internal jugular or lower extremity venous catheter placement or exchange. Over longer periods of time, filter struts may erode through the vessel wall and can penetrate adjacent structures such as the duodenum, aorta, or vertebral bodies. Finally, IVC thrombosis is one of the major risks of long-term IVC filter placement and this risk increases the longer the filter remains in place. One study has shown a 33% incidence of IVC thrombosis after 8 years with some filters.^18^ Among retrievable filters, the rate of IVC thrombosis is much lower and estimated to be between 0.6% and 8%.^25^ IVC thrombosis may result in further lower extremity venous obstructive complications. Discussing these risks regarding filter placement can often provoke anxiety for the patient, but it should be affirmed that the benefit still outweighs the risks in patients who cannot tolerate anticoagulation.

## Types of Filters

The first percutaneous IVC filter placed in 1969 was a Greenfield filter, which is a conical permanent filter. Over the years, several design variations of IVC filters have been developed to improve the prevention of PE while lowering risk of caval thrombosis and facilitating future removal. The mechanics of the conical-shaped filters have been well documented for being effective in thrombus capture while minimizing flow impedance, although hexagonal and “bird’s nest” filters exist as well.

Four major classifications of IVC filters include (1) permanent filters, which are intended for long-term attachment to the IVC wall; (2) temporary filters, which are not attached to the IVC wall but suspended endovascularly then attached to the skin/subcutaneous tissue; (3) convertible filters, which are permanent filters with a retrievable filter attachment; and (4) retrievable filters that are equipped with barbs or hooks and can be removed.^13^ Retrievable IVC filters are most used in practice today as the long-term presence of IVC filters has been associated with increased risk of recurrent DVT.^14^

A few major IVC filters used in the US are summarized in Table 2. Most available filters are similarly effective in preventing PE, but they differ in complication type and rate. Greenfield filters are the standard for comparison due to their long track record of long-term patency and low risk of thrombosis. However, they are permanent filters and require a rather large 12F delivery system. The Bard G2 Filter (Bard Peripheral Vascular, Inc.) is a retrievable filter associated with high technical success with longer indwelling times but also is associated with higher rates of fracture and migration. The Cook Celect (Cook Medical) is a newer version of the Cook Gunther Tulip designed to reduce tilt and fluoroscopy time with the use of platinum markers. The OptEase Filter (Cordis) is unique in its hexagonal design and caudal hook, which allows for retrieval via a femoral approach. The Argon Option Filter (Argon Medical Devices) is delivered via a 5F system, the smallest on the market.^26,27^ Bird’s nest filters can be used for IVCs that are more than 30 mm in diameter.

**Table 2:** Commonly used inferior vena cava (IVC) filters in the United States.


FILTER	COMPANY	FEATURES

Greenfield filters	Boston Scientific	Long-term patency, low risk of thrombosis, 12F delivery system

Bard G2 filter	Bard Peripheral Vascular, Inc.	High technical success, longer indwelling time, but higher rates of fracture and migration

Cook Gunther Tulip	Cook Medical	One of the first IVC filters approved by the U.S. Food and Drug Administration for retrieval

Cook Celect	Cook Medical	Newer version of Cook Gunther Tulip, designed to reduce tilt and fluoroscopy time

OptEase Filter	Cordis	Unique hexagonal design and caudal hook that allows for retrieval via femoral approach

Argon Option Filter	Argon Medical Devices	Smallest on the market with a 5F delivery system


## Removal Techniques

IVC filters were once considered permanent devices that remained throughout the patient’s lifetime. In 2010, the FDA announced a new recommendation to remove filters once they are no longer needed.^28^ This has resulted in increased rates of IVC filter removal and, consequently, the development of various techniques for removal. Permanent filters are generally not amenable to endovascular retrieval, whereas retrievable filters designed for removal often have a neck or hook that allows for snaring from above and/or below. Over time, these necks or hooks can often embed in the venous wall, causing filter tilting away from the central axis of the vena cava, protrusion from the vena cava, or even fracture, and thus creating a much more difficult endovascular removal.

We briefly cover some commonly used techniques for difficult filter removal—or those that have failed standard attempts with snare and sheath. Tilted filters embedded into the caval wall can be challenging to retrieve. As a first attempt, a curved inner sheath can be used to add directionality to the snare. If this fails, a curved catheter can be used to pass a wire underneath the filter legs and back cephalad. The wire is then snared via the same sheath access, allowing for a sheath to be advanced over the filter for removal. This technique is known as the “loop-snare” method.^29^ During the loop step, this method is often aborted due to fear of leg deformity or fracture. In cases where the hook of the filter is embedded in the IVC wall, a modified loop-snare technique also known as the “hangman” can be performed. This occurs when a loop-snare is formed between the neck of the filter and the IVC wall, allowing for either filter detachment from the fibrin encasement and/or a sheath to be passed onto the hook of the filter for removal.^29^

The use of rigid endobronchial forceps can be used to grasp the hook once it is freed from the surrounding fibrin. Then, the sheath is advanced over the forceps and onto the hook to capture the filter.^29^ The third most common technique and more often used by interventionalists is the use of a photothermal laser to ablate the encasing fibrin sheath, followed by snaring of the filter. Ultimately, open surgical retrieval with primary repair of the IVC is necessary in some cases. With the advent of robotic surgery, laparoscopic robotic-assisted IVC filter removal also has proven to be a safe and efficacious strategy.

## Conclusion

IVC filters are a safe and effective treatment for preventing clinically significant PE in patients who cannot tolerate anticoagulation. Over the years, several designs have been developed with unique risks and benefits that are crucial for surgeons and interventionalists to understand and consider depending on patient needs. IVC filters should be closely followed and promptly removed to prevent future morbidities. Considering that IVC filters will remain a mainstay of treatment, advancing the toolbox of filter removal techniques, including robotic-assisted removal in those not amenable to endovascular intervention should be at the forefront of future discussion.

## Key Points

- Though recommendations regarding specific patient scenarios differ between professional organizations, indications for inferior vena cava (IVC) filter placement generally include patients who have venous thromboembolism and have contraindications to and/or have failed anticoagulation therapy.
- Several filters have been created to optimize thrombus capture while allowing for future retrieval and prevention of filter thrombosis, which is a major complication of IVC filters present for an extended duration.
- Retrievable IVC filters in place for an extended duration often become difficult to remove through standard methods. Innovative retrieval techniques can be used to safely and effectively remove filters with minimal risk to the patient.
